# Microstructural differences in the cingulum and the inferior longitudinal fasciculus are associated with (extinction) learning

**DOI:** 10.1186/s40359-024-01800-y

**Published:** 2024-06-03

**Authors:** Alina Nostadt, Lara Schlaffke, Christian J. Merz, Oliver T. Wolf, Michael A. Nitsche, Martin Tegenthoff, Silke Lissek

**Affiliations:** 1 Department of Neurology, BG University Hospital Bergmannsheil, Ruhr University Bochum, Bochum, 44789 Germany; 2 Ruhr University Bochum, Bochum, Germany; 3 Department of Cognitive Psychology, Institute of Cognitive Neuroscience, Faculty of Psychology, Ruhr University Bochum, Bochum, 44801 Germany; 4 Leibniz Research Centre for Working Environment and Human Factors, Department of Psychology and Neurosciences, Dortmund, 44139 Germany; 5 German Centre for Mental Health (DZPG), Bochum, Germany; 6 University Hospital OWL, Protestant Hospital of Bethel Foundation, University Clinic of Psychiatry and Psychotherapy and University Clinic of Child and Adolescent Psychiatry and Psychotherapy, Bielefeld University, Bielefeld, 33617 Germany

**Keywords:** Diffusion tensor imaging, Cingulum, Inferior longitudinal fasciculus, Associative learning, Extinction learning, Renewal effect

## Abstract

Cognitive functions, such as learning and memory processes, depend on effective communication between brain regions which is facilitated by white matter tracts (WMT). We investigated the microstructural properties and the contribution of WMT to extinction learning and memory in a predictive learning task. Forty-two healthy participants completed an extinction learning paradigm without a fear component. We examined differences in microstructural properties using diffusion tensor imaging to identify underlying neural connectivity and structural correlates of extinction learning and their potential implications for the renewal effect. Participants with good acquisition performance exhibited higher fractional anisotropy (FA) in WMT including the bilateral inferior longitudinal fasciculus (ILF) and the right temporal part of the cingulum (CNG). This indicates enhanced connectivity and communication between brain regions relevant to learning and memory resulting in better learning performance. Our results suggest that successful acquisition and extinction performance were linked to enhanced structural connectivity. Lower radial diffusivity (RD) in the right ILF and right temporal part of the CNG was observed for participants with good acquisition learning performance. This observation suggests that learning difficulties associated with increased RD may potentially be due to less myelinated axons in relevant WMT. Also, participants with good acquisition performance were more likely to show a renewal effect. The results point towards a potential role of structural integrity in extinction-relevant WMT for acquisition and extinction.

**Supplementary Information:**

The online version contains supplementary material available at 10.1186/s40359-024-01800-y.

## Introduction

Learning and memory rely on the communication between brain areas that are functionally involved during these processes [1, 2]. Efficient communication between different brain regions is essential for the brain’s ability to encode new information and recall memories [3]. Therefore, cognitive functions such as learning and memory processes require information transmission via white matter tracts (WMT) in the brain [4–7]. WMT are myelinated neuronal fibers that enable the transmission of electrical signals and the integration of information across different brain regions which is essential for the structural basis of human behavior [7, 8]. The microstructural organization of WMT determines the integrity and connectivity of these tracts and therefore can influence learning and memory [5, 9–11]. Studies have shown that specific WMT play a crucial role during extinction learning [12–14], which is a form of learning that refers to the process by which a conditioned response gradually diminishes when it is no longer reinforced [15–17]. During extinction learning and the recall of extinction memory, complex neural circuits and connections between specific brain areas, such as the prefrontal cortex (PFC) and hippocampus (HC), are functionally involved [17–21]. It is assumed that during extinction, previously learned associations are not erased, but rather new associations are formed as well as novel pathways and connections are built [22, 23]. As a result of neuroplasticity, the modification of neural connections leads to changes in brain structure and microstructural properties of WMT [24, 25].

Several white matter pathways are associated with learning and memory processes such as the uncinate fasciculus (UNC), inferior longitudinal fasciculus (ILF), temporal and superior part of cingulum (CNG), inferior fronto-occipital fasciculus (IFOF), and fornix (FX) [12, 26–28]. Alterations of these connections can result in impairments of cognitive functions and may be therefore of great interest for extinction learning and renewal [29–31]. For example, the UNC, which connects parts of the temporal lobe and PFC, was shown to be involved during extinction and conditioning paradigms [32, 33]. The ILF connects the occipital and temporal lobes and plays a crucial role in visual processing and object recognition [34]. Its integrity and connectivity are essential for transferring and integrating visual information, contributing to perception, and understanding of the visual world [34]. The CNG is involved in a wide range of cognitive functions, including attention, emotion regulation, decision-making, and memory [35]. The cingulate gyrus has connections with structures of the limbic system, such as the hippocampus and amygdala, as well as with the prefrontal cortex [35]. The IFOF is a large WMT that reaches from the occipital lobe to the PFC and is associated with semantic language processing and goal-oriented behavior [36]. The FX is part of the limbic system and the major tract of the HC. Disruptions of the FX can cause memory loss and other impairments of cognitive functions [37].

In this study, we investigated the microstructural properties of WMT involved in extinction learning and memory recall by means of DTI. Diffusion-weighted imaging (DWI) data were acquired from healthy participants, which performed an extinction learning paradigm without a fear component. We aimed to gain insights into the contributions of white matter pathways to extinction learning and memory during a predictive learning task based on associative learning. We assume that differences in microstructural properties may reveal structural correlates of learning and extinction memory recall, such as better structural connectivity, i.e., connectivity with more directed diffusion, for participants with enhanced learning performance [38]. By studying the microstructural properties and connectivity of relevant brain regions, we can broaden our understanding of the neural basis of extinction and its potential relevance to psychiatric disorders and the renewal effect.

## Methods

This study applies methods identical to or resembling those used in our previous publications [11, 18, 39–41]. Therefore, some descriptions of these methods here have been adapted from those sources.

### Participants

We acquired DWI imaging data from 45 healthy, right-handed (Edinburgh Handedness Inventory) volunteers, excluded 3 participants from further analysis due to insufficient data quality, and conducted the analysis with 42 participants (25 females) with an age range of 20–35 years (mean 26.14 +/- 3.34 years). The DWI data were collected as part of an fMRI study where we recruited volunteers to be a part of a study with two treatment groups. Participants were randomly assigned to the treatment groups and received either 20 mg hydrocortisone (*n* = 23, 13 females) or placebo (*n* = 19, 12 females) before the start of the experiment. DWI data were collected directly after the participants finished the predictive learning task, approximately 2 h after intake of hydrocortisone/placebo. We expected no significant cortisol-induced short-term microstructural changes [42–44], but we controlled for possible confounding effects with the assigned treatment group as a covariate of interest.

### Predictive learning task

**Fig. 1:**
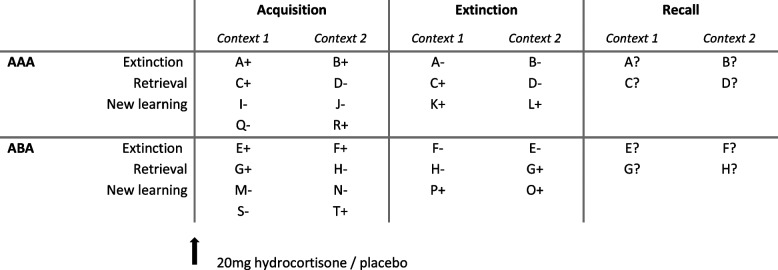
Schematic overview of the predictive learning task. Participants learned, extinguished, and recalled stimulus-outcome associations during three different task phases. Before the acquisition, we administered either 20 mg hydrocortisone or placebo. Appetitive food stimuli were presented and participants had to predict whether the food served in one out of two restaurants (context 1, context 2) will cause stomach ache or not. The characters reflect the food stimulus and +/- indicates the related consequence (+: no stomach ache, -: stomach ache). During acquisition and extinction phases, participants received feedback after their response if their answer was correct. Extinction stimuli changed their consequence during the extinction phase while retrieval stimuli did not. *AAA*: Stimuli during the extinction phase were presented in the same restaurant (context). *ABA*: Stimuli during the extinction phase were presented in a different restaurant (context). New learning stimuli were introduced to balance the design. In the recall phase, participants had to predict again the consequence of the presented food stimulus but received no feedback. The letters indicate the different food stimuli that we presented during each trial. This task design and schematic overview is similar to our previous studies which is reported in more detail in Lissek et al. [39, 40]; Lissek, Klass, and Tegenthoff  [41]

Participants performed a predictive learning task that was designed to investigate extinction learning and recall which has already been used and described in previous studies, therefore some text passages in this paragraph were recycled from those sources [17, 18, 39, 40, 45, 46]. During three subsequent task phases, participants learned, extinguished, and recalled stimulus-outcome associations. In the first learning phase (acquisition), participants learned to associate a food item that was presented in one of two contexts (context 1 or 2, see Fig. 1) with a particular consequence (food causes stomach ache or not). During the extinction learning phase, some of the previously learned stimulus-outcome associations were extinguished (extinction stimuli – consequence change). Half of the extinction stimuli were presented again in context 1, as during acquisition (acquisition in context 1 (A), extinction in context 1 (A), recall in context 1 (A) ◊ Extinction AAA - condition without context change), and the other half in a different context 2 (acquisition in context 1 (A), extinction in context 2 (B), recall in context 1 (A) ◊ Extinction ABA - condition with context change) in randomized order. In addition, new learning stimuli were introduced, replacing those presented during acquisition which were not presented again (new learning). Furthermore, for other stimuli, the consequence did not change (retrieval stimuli). These two types of stimuli served as a control for learning success.

In the test phase (recall), extinction and retrieval stimuli were presented again in their acquisition contexts. In this study, we focus on acquisition and extinction stimuli during extinction learning and recall. In the recall phase, we tested the memory performance and especially context-dependent extinction memory. If participants responded during the condition ABA using the associations learned during acquisition, they showed context-dependent renewal. With the exception that during the recall phase participants received no feedback at all, trials were identical to those during acquisition. For a schematic overview of the task design see Fig. 1. For a more detailed description of the predictive learning task [39, 40] and its trial structure see [41].

### Behavioral data analysis

Due to the reuse of the predictive learning task, that was already applied in its current or modified version in our previous studies, we used parts of the methodology for behavioral data analysis which are described in more detail in Lissek et al. [18, 40]; Lissek, Klass, and Tegenthoff [41]. For behavioral data analysis, we used the statistical software Matlab (V.2022b, The Math Works, USA). For every single participant, we calculated the mean percent error rates of learning and memory recall performance for the different learning phases and experimental conditions. Errors in acquisition and extinction learning were defined as responses stating the incorrect association between the context-cue-compound and the consequence. During recall, we tested the correctness of response for retrieval stimuli (in both, context change and no context change) as well as the extinction memory. For extinction stimuli, we were interested in the context-dependent recall of extinction memory (i.e., the renewal effect) and the recall of AAA extinction memory in the experimental condition without context change. In case of renewal, associations learned during acquisition in context A should reappear in the recall phase, which is again performed in context A, while extinction was performed in context B (ABA condition). In contrast, the AAA condition constitutes a control condition for context-dependent extinction learning, since here all learning phases are performed in an identical context. We calculated the mean percentage of renewal responses from participants if they had at least 20% renewal responses during the recall of ABA extinction memory. 0 − 20% renewal responses are stated as no renewal. If extinction learning is successful, responses during the recall phase should reflect the associations learned during extinction. We calculated the mean and standard deviation of each participant’s learning errors in each learning phase and experimental condition. To further analyze the renewal performance and reveal possible coherences with the acquisition performance, we assigned each participant to one of three groups based on their learning performance (low error (LE): 0% – 12,5% errors, medium error (ME): 12,5 − 25% errors, high error (HE): 25 − 50%). We calculated the median and interquartile range (IQR) to analyze the learning error rates on a group level.

### Imaging data

Brain imaging data were obtained using a Philips Achieva 3.0 T X-Series MR scanner (Philips, The Netherlands) and a 32-channel SENSE head coil. This data acquisition and analysis protocol was similar to our previous study (for details see Schlaffke et al. 2017 [11]). In brief, DWI was performed using 60 diffusion-weighted gradient orientations (b = 1000 s/mm^2^) with 6 interleaved non-diffusion weighted images resulting in 60 parallel acquired slices. For all gradient orientations, two volumes were acquired and averaged for a better SNR resulting in a total acquisition time of approximately 10 min. The following acquisition parameters were used: FOV = 224 × 224 × 120 mm^3^, 2 × 2 × 2 mm^3^ voxel size, resulting in 60 slices, TR 8151 ms, TE 88 ms. We acquired a high resolution (1 × 1 × 1 mm^3^ voxel size) T1-weighted image as a reference for EPI-distortion correction using the following parameters: TR = 8.3 ms, TE = 3.8 ms Flip angle 9◦, FOV 256 × 256 × 220 mm^3^.

### DTI data processing and tractography

The DWI data were analyzed with the Explore DTI toolbox(Leemans et al. 2009) for Matlab (V.2022b, The Math Works, USA). For pre-processing, we corrected for subject motion and eddy current induced geometric distortions, as well as for EPI distortions (Leemans et al. 2009). After tensor estimation (Leemans et al. 2009), we performed a whole-brain fiber tractography [47, 48]: we used a seed resolution grid of 2 × 2 × 2 mm^3^ and the following tracking stop criteria: 0.2 minimum fractional anisotropy (FA), 30° angle threshold, and 2 mm step size (Schlaffke et al. 2017 [11]). To select the white matter pathways of interest (bilateral): ILF, superior and temporal part of the CNG, IFOF, UNC, and FX, we used a region of interest (ROI) approach as described in Catani & Schotten [49]. We applied AND and NOT ROIs manually to extract the WMT of interest for every single subject. Next, we calculated λ_1−3_, the mean FA, radial diffusivity (RD) and the mean diffusivity (MD) of the selected tracts. To investigate the relation between the structural organization of the WMT and learning and memory performance (percent error rate), we used Spearman rank correlations.

## Results

### Behavioral data

Participants had a median (IQR) of 16.41% (10.94) learning errors during the acquisition phase. In the extinction learning phase, participants showed a median of 18.75% (12.5) learning errors for ABA extinction stimuli and 18.75% (12.5) learning errors for AAA extinction stimuli. During the recall phase, we observed 0% (60) ABA renewal responses and 0% (20) recall errors for AAA extinction stimuli. There was no main effect of treatment (one-way ANOVA, *p* > 0.1) on behavioral performance during all task phases (acquisition, extinction, renewal).

First, we tested for the main effects of acquisition and Extinction ABA condition errors and interaction effects (acquisition*Extinction ABA) on renewal responses. There was a main effect of acquisition errors on renewal responses (two-way ANOVA F(2) = 3.16, *p* = 0.05), but no main (two-way ANOVA, F(2) = 2.58, *p* = 0.09) or interaction effect (two-way ANOVA, F(4) = 0.38, *p* = 0.8) of Extinction ABA errors. As a planned comparison, we calculated a correlation between the mean percent acquisition errors and the mean percent renewal responses (Spearman correlation: *r* = − 0.09, *p* = 0.5), showing that acquisition learning errors do not correlate with the mean percentage of renewal responses.

To investigate if the significant main effect of acquisition errors on renewal responses varied for groups with different learning performances, we compared the total number of participants who showed renewal responses across three subgroups. We subdivided the participants into three subgroups based on their learning performance (low, medium, high) in acquisition (LE: *n* = 13, ME: *n* = 19, HE: *n* = 10). Next, we calculated the participants with renewal responses within the subgroups. In the HE group were fewer participants who showed renewal (HE renewal: *n* = 1 of 10, 10%) compared to low (LE renewal: *n* = 5 of 12, 41.6%) and medium error groups (ME renewal: *n* = 9 of 19, 47.37%). HE vs. ME: X^2^ = 12.8, *p* < 0.01; HE vs. LE: X^2^ = 2.75, *p* = 0.09; ME vs. LE: X^2^ = 5.51, *p* = 0.01.

### Microstructural properties of WMT and learning performance

**Fig. 2:**
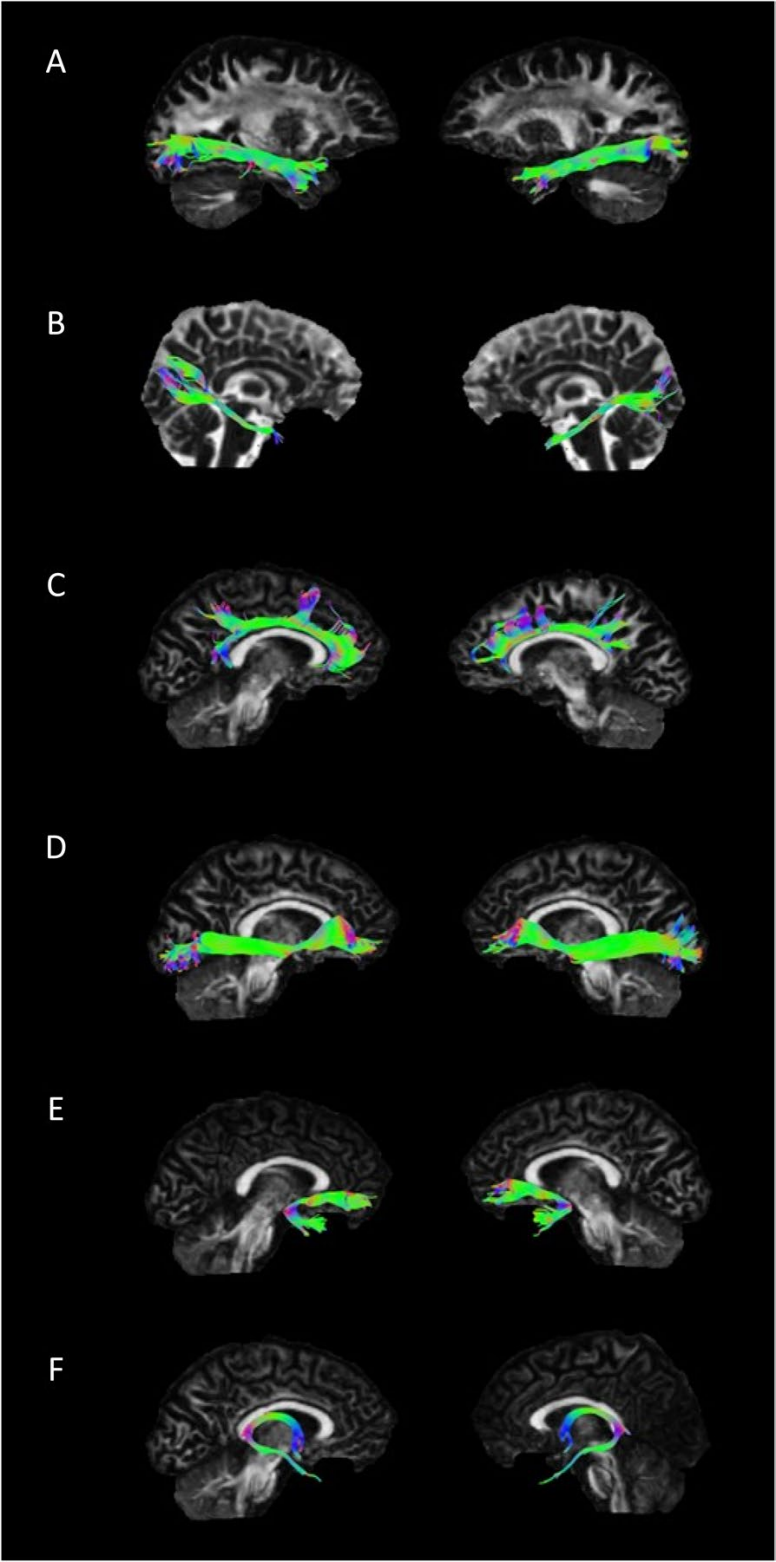
An example from one participant depicting the reconstruction of the WMT of interest. For every participant, we extracted the left and right (**A**) ILF (**B**) the temporal part of CNG, **C** the superior part of CNG, **D** IFOF, **E** UNC, and **F** FX

See Fig. 2 for an overview of our WMT of interest and Supplement 1. of the respective microstructural properties (mean and SD: λ1, λ2, λ3, FA, RD, MD). Based on our hypothesis and the behavioural results, which have demonstrated a possible coherence between participants with high acquisition errors and lower renewal, we tested for main and interaction effects of acquisition learning errors and renewal performance on mean FA, RD, and MD of WMT of interest (two-way ANOVA).

 FA: There was a main effect of acquisition learning errors on mean FA of the left ILF (F(1) = 5.12, *p* = 0.02), right ILF (F(1) = 4.09, *p* = 0.05), and right temporal CNG (F(1) = 6.53, *p* = 0.01)). We observed no interaction effect of acquisition learning errors*renewal responses (*p* > 0.05), and all other WMT had no main or interaction effects (*p* > 0.05) on mean FA. Also, no main effect of treatment on the mean FA of WMT was observed (one-way ANOVA, *p* > 0.05). RD: We observed a main effect of acquisition learning errors on mean RD of the left ILF (F (1) = 4, *p* = 0.05), right ILF (F(1) = 3.77, *p* = 0.05), and right temporal CNG (F(1) = 5.96, *p* = 0.01). There was no interaction effect of acquisition learning errors*renewal responses (*p* > 0.05), and all other WMT had no main or interaction effects (*p* > 0.05) on mean RD. Also, no main effect of treatment on the mean RD of WMT was observed (one-way ANOVA, *p* > 0.05). MD: There was a main effect of acquisition learning errors on right temporal CNG (F(1) = 4.28, *p* = 0.04), and no main effect of treatment on the mean MD of WMT was observed (one-way ANOVA, *p* > 0.05).

**Fig. 3:**
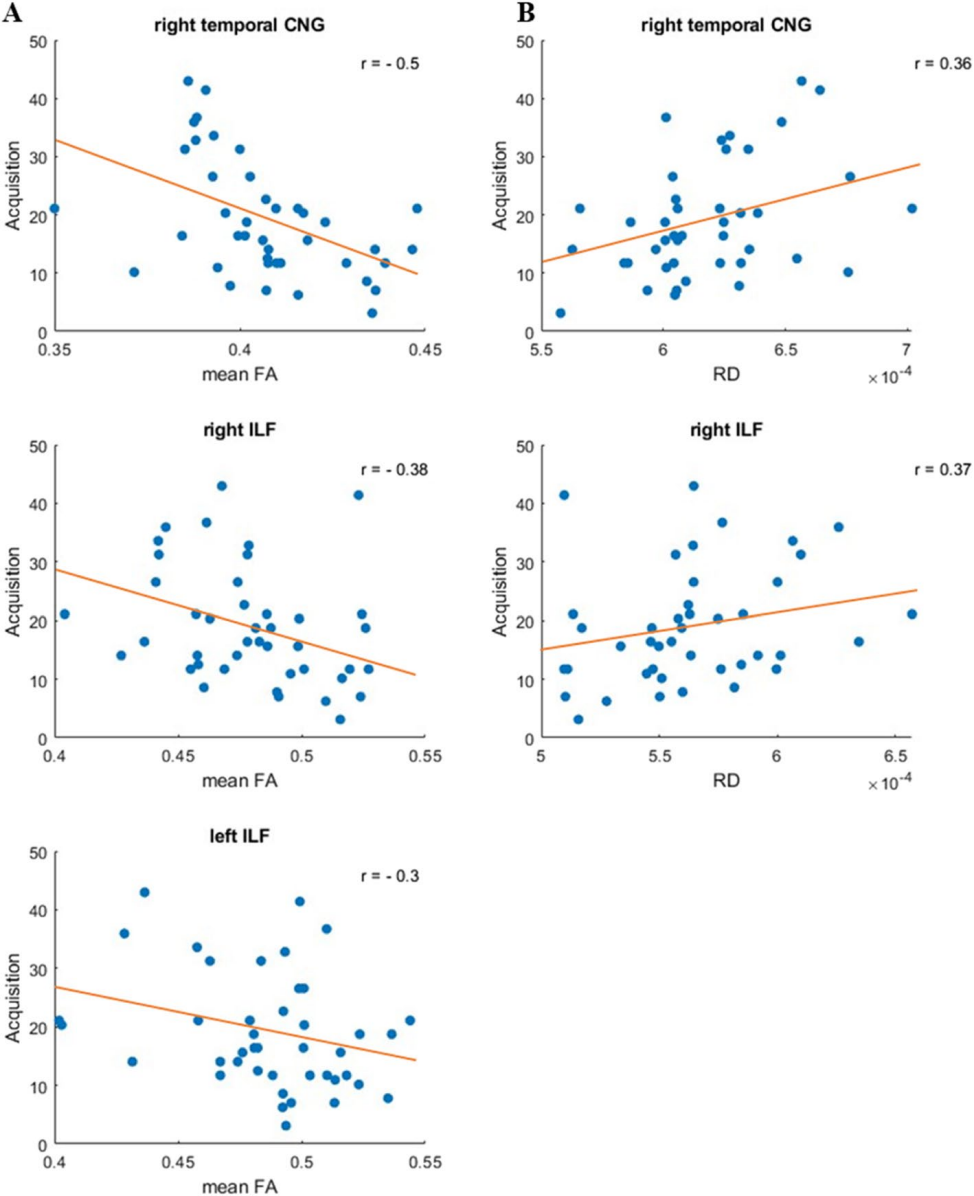
**A** Negative correlation of acquisition errors and mean FA of right temporal CNG (*r* = -0.5, *p* < 0.001). right ILF (*r* = -0.38, *p* = 0.01), and left ILF (*r* = -0.3, *p* = 0.05). **B** Positive correlation of acquisition errors and mean RD of right temporal CNG (*r* = 0.36, *p* = 0.01), and right ILF (*r* = 0.37, *p* = 0.01)

We hypothesized, that enhanced structural connectivity reflected by differences in microstructural properties of WMT of interest would correlate with learning performance during the predictive learning task. To further investigate the correlation between microstructural properties of the WMT of interest, and the learning and memory performance, we correlated behavioral performance (acquisition errors and the renewal rate) with the microstructural properties (FA, RD, MD) of the WMT of interest (Spearman correlation). Results showed negative correlations between acquisition errors and FA, as well as positive correlations with RD, for various WMT. For an overview of the significant correlations with mean FA and mean RD (see Fig. 3). In addition, a planned contrast (Spearman correlation) revealed a positive correlation between the mean MD of right temporal CNG and acquisition learning errors (*r* = 0.3, *p* = 0.03).

## Discussion

We analyzed microstructural properties of WMT that are assumed to be functionally involved in learning and memory formation in an extinction learning paradigm. We found negative correlations between errors made during acquisition learning and the mean FA of specific WMT, including the right and left ILF and the temporal part of the CNG. The FA is used as a measure of connectivity in the brain by examining the orientation and magnitude of water movement in the tissue and so providing information about the microstructural organization and integrity of white matter tracts [50]. Higher FA values suggest better connectivity (connectivity with more directed diffusion) and in addition, facilitate communication between brain areas important for learning and memory [26, 50].

In our study, we revealed a negative correlation between acquisition learning errors and mean FA of bilateral ILF and right temporal CNG. Participants who performed well during acquisition learning (indicated by low error rates) had higher FA values in these WMT. This suggests that participants who performed well in acquisition had enhanced structural connectivity in the WMT involved in learning and memory processes (such as the bilateral ILF and the right temporal part of the CNG). The involvement of ILF and the temporal part of the CNG is in line with previous research [34], as ILF connects the occipital and temporal lobes and is important for object recognition and visual information processing. The CNG is thought to be involved in attention and decision-making and especially the temporal part of the CNG is associated with the hippocampal memory network and therefore relevant for memory retrieval [35]. Our results complement these findings by highlighting the role of the temporal part of the CNG, which connects the temporal lobe and the hippocampal area with other brain regions, thus potentially improving learning performance, which may result in lower error rates.

For the right ILF and the right temporal part of the CNG, we additionally found a positive correlation between RD and acquisition errors. Differences in RD are associated with alterations in the microstructure of WMT. An increase in RD suggests changes in the microstructure of white matter tracts, such as less myelinated axons, which can lead to cognitive impairments [51, 52]. In our study, participants who did not perform well during acquisition (high frequency of acquisition errors) showed lower mean FA and higher RD in the right ILF and right temporal part of the CNG, indicating changes or less myelinated axons in WMT of interest accompanied by cognitive deficits such as difficulties during acquisition reflected by higher error rates. In addition, acquisition error rates positively correlated with the MD of the right temporal CNG. We assume that this finding is due to the decrease of the tensor diameter (RD) and does not reflect that differences in MD of this WMT result in cognitive impairments such as higher learning errors.

There were differences in the recall of extinction memory (renewal performance) between participants with low, medium, and high acquisition errors. Participants in the low and medium error groups showed more renewal responses compared to the high error group. We assume that participants with a good acquisition performance were more likely to recall stimulus-outcome associations from the initial learning phase (acquisition) because these associations were consolidated to a larger degree. In line with our hypothesis, our findings suggest that improved renewal performance in our extinction learning task depends on acquisition performance which correlates with structural connectivity in WMT of interest, as described.

We investigated the microstructural properties of WMT involved in learning and memory during an extinction learning paradigm. We found that FA, a measure of connectivity and microstructural integrity, was associated with acquisition learning errors. Participants with better acquisition performance (lower error rates) had higher FA values in specific white matter tracts, including the bilateral ILF and the temporal part of the CNG, indicating improved connectivity and communication between brain areas relevant for learning and memory. This suggests that good acquisition and extinction memory performance are linked to structural connectivity in white matter tracts, such as the ILF and the temporal part of the CNG, which are associated with the hippocampal memory network, visual processing, and decision-making. Additionally, our investigation of RD revealed that participants with poor acquisition performance had lower FA and higher RD in the right ILF and right temporal part of the CNG. This suggests possible less myelinated axons and microstructural differences in these white matter tracts, which may contribute to learning difficulties associated with increased RD. The experimental design of this study cannot account for intra-individual learning-related changes in the microstructural properties of WMT. A modified experimental design with a DWI data acquisition before and after the learning task could be applied to address this question. Given the relatively small sample size, potentially restricting the generalizability of the findings, a subsequent study employing a larger sample size could be pursued. Although no influence of the pharmacological intervention on microstructural characteristics of the WMT were expected and not found during the analyses, in a subsequent study, the intervention might be excluded.

## Conclusion

Our findings emphasize the importance of white matter microstructural properties in learning and memory processes. Specifically, the positive association between acquisition learning performance and high FA in WMT that are relevant for object recognition and memory recall. It is suggested that structural connectivity plays also a role in renewal performance within this predictive learning task. Our results are in line with studies that highlight the importance of the acquisition phase in the recall of extinction memory and renewal [39]. Also, the results of the present study suggest that poor acquisition learning performance is associated with less myelinated axons as indicated by a high RD in right ILF and right temporal CNG. The HE group, which had poor acquisition performance, exhibited the lowest amount of renewal responses compared to participants with medium and low acquisition errors. This indicates that associations that are poorly learned during acquisition are presumably unstable and may therefore lead to superior extinction associations during recall, and less renewal. In order to obtain a more comprehensive understanding of the communication between the brain regions of interest and the neural mechanisms involved in the learning and recall of extinction memory, it would be beneficial to investigate also task-related functional connectivity between these brain areas.

### Supplementary Information

Supplementary Material 1.

## Data Availability

Data can be made available upon reasonable request with the need for a formal data sharing agreement. Please contact the corresponding author (Alina Nostadt) regarding data.

## References

[1] Thompson RF, Kim JJ. Memory systems in the brain and localization of a memory. Proc Natl Acad Sci U S A. 1996;93(24):13438–44. 10.1073/pnas.93.24.13438 PMC33628 8942954

[2] Ackerman S (1992). Discovering the brain. Discovering Brain.

[3] Bonnefond M, Kastner S, Jensen O. Communication between brain areas based on nested oscillations. eNeuro 2017:4;NEURO.0153-16.2017. 10.1523/ENEURO.0153-16.2017 PMC5367085 28374013

[4] Horne A, Ding J, Schnur TT, Martin RC (2022). White Matter correlates of Domain-Specific Working Memory. Brain Sci.

[5] Dziemian S, Appenzeller S, von Bastian CC, Jäncke L, Langer N. Working Memory Training effects on White Matter Integrity in Young and older adults. Front Hum Neurosci 2021:15;605213. 10.3389/fnhum.2021.605213 PMC8079651 33935667

[6] Bouyeure A, Bekha D, Patil S, Hertz-Pannier L, Noulhiane M. Maturity of white matter tracts is associated with episodic memory recall during development. Cereb Cortex Commun 2022;3:tgac004. 10.1093/texcom/tgac004 PMC8895309 35261977

[7] Filley CM, Fields RD. White matter and cognition: Making the connection. J Neurophysiol. 2016:116; 2093–2104 Preprint at 10.1152/jn.00221.2016. 10.1152/jn.00221.2016 PMC5102321 27512019

[8] Bassett DS, Brown JA, Deshpande V, Carlson JM, Grafton ST. Conserved and variable architecture of human white matter connectivity. NeuroImage. 2011;54:1262–79. 10.1016/j.neuroimage.2010.09.006 20850551

[9] Jolles D (2016). Plasticity of left perisylvian white-matter tracts is associated with individual differences in math learning. Brain Struct Funct.

[10] Taubert M (2010). Dynamic properties of human brain structure: learning-related changes in cortical areas and associated fiber connections. J Neurosci.

[11] Schlaffke L, Leemans A, Schweizer LM, Ocklenburg S, Schmidt-Wilcke T (2017). Learning morse code alters microstructural properties in the inferior longitudinal fasciculus: a DTI study. Front Hum Neurosci.

[12] Hermann A, Stark R, Blecker CR, Milad MR, Merz CJ (2017). Brain structural connectivity and context-dependent extinction memory. Hippocampus.

[13] Fani N (2015). Fear-potentiated startle during extinction is associated with white matter microstructure and functional connectivity. Cortex.

[14] Nees F (2019). White matter correlates of contextual pavlovian fear extinction and the role of anxiety in healthy humans. Cortex.

[15] Bouton ME, Bolles RC (1979). Role of conditioned contextual stimuli in reinstatement of extinguished fear. J Exp Psychol Anim Behav Process.

[16] Bouton ME, Bolles RC (1979). Contextual control of the extinction of conditioned fear. Learn Motiv.

[17] Lissek S, Klass A, Tegenthoff M. Left Inferior Frontal Gyrus participates in mediating the Renewal Effect irrespective of Context Salience. Front Behav Neurosci 2020;14:43. 10.3389/fnbeh.2020.00043 PMC7118360 32292332

[18] Lissek S, Glaubitz B, Uengoer M, Tegenthoff M (2013). Hippocampal activation during extinction learning predicts occurrence of the renewal effect in extinction recall. NeuroImage.

[19] Sotres-Bayon F, Diaz-Mataix L, Bush DEA, LeDoux JE (2009). Dissociable roles for the ventromedial prefrontal cortex and amygdala in fear extinction: NR2B contribution. Cereb Cortex.

[20] Gass JT, Chandler LJ (2013). The plasticity of extinction: contribution of the Prefrontal Cortex in treating addiction through inhibitory learning. Front Psychiatry.

[21] Quirk GJ, Mueller D. Neural mechanisms of extinction learning and retrieval. Neuropsychopharmacology. 2008;33:56–72 Preprint at 10.1038/sj.npp.1301555 . 10.1038/sj.npp.1301555 PMC2668714 17882236

[22] Myers KM, Davis M (2002). Behavioral and neural analysis of extinction. Neuron.

[23] Delamater AR (2004). Experimental extinction in pavlovian conditioning: behavioural and neuroscience perspectives. Q J Experimental Psychol Sect B: Comp Physiological Psychol.

[24] Frizzell TO et al. White Matter Neuroplasticity: Motor Learning activates the Internal Capsule and reduces hemodynamic response variability. Front Hum Neurosci 2020;4:509258. 10.3389/fnhum.2020.509258 PMC7649291 33192383

[25] Frizzell TO (2022). Imaging functional neuroplasticity in human white matter tracts. Brain Struct Funct.

[26] Vestergaard M (2011). White Matter Microstructure in Superior Longitudinal Fasciculus Associated with spatial Working Memory performance in children. J Cogn Neurosci.

[27] Salminen T, Mårtensson J, Schubert T, Kühn S (2016). Increased integrity of white matter pathways after dual n-back training. NeuroImage.

[28] Dziemian S, Appenzeller S, von Bastian CC, Jäncke L, Langer N. Working Memory Training effects on White Matter Integrity in Young and older adults. Front Hum Neurosci 2021;15:605213. 10.3389/fnhum.2021.605213 PMC8079651 33935667

[29] Chang YL, Chao RY, Hsu YC, Chen TF, Tseng W (2021). Y. I. White Matter network disruption and cognitive correlates underlying impaired memory awareness in mild cognitive impairment. Neuroimage Clin.

[30] Qiao Y et al. The associations between White Matter disruptions and Cognitive decline at the early stage of subcortical vascular cognitive impairment: a case–control study. Front Aging Neurosci 2021;13:681208. 10.3389/fnagi.2021.681208 PMC8364958 34408641

[31] Sweeney JA (2016). White Matter Abnormalities in post-traumatic stress disorder following a specific traumatic event. EBioMedicine.

[32] Costanzo ME (2016). White matter microstructure of the uncinate fasciculus is associated with subthreshold posttraumatic stress disorder symptoms and fear potentiated startle during early extinction in recently deployed service members. Neurosci Lett.

[33] Hölzel BK et al. Mindfulness-based stress reduction, fear conditioning, and the Uncinate Fasciculus: a pilot study. Front Behav Neurosci 2016;10;124. 10.3389/fnbeh.2016.00124 PMC4908122 27378875

[34] Herbet G, Zemmoura I, Duffau H. Functional Anatomy of the Inferior Longitudinal Fasciculus: From Historical Reports to Current Hypotheses. Front Neuroanatomy 2018;12 Preprint at 10.3389/fnana.2018.00077. 10.3389/fnana.2018.00077 PMC6156142 30283306

[35] Bubb EJ, Metzler-Baddeley C, Aggleton JP. The cingulum bundle: Anatomy, function, and dysfunction. Neurosci Biobehav Rev. 2018;92:104–127 Preprint at 10.1016/j.neubiorev.2018.05.008 . 10.1016/j.neubiorev.2018.05.008 PMC6090091 29753752

[36] Conner AK (2018). A connectomic atlas of the human Cerebrum-Chap. 13: Tractographic description of the Inferior Fronto-Occipital Fasciculus. Operative Neurosurg.

[37] Nowrangi MA, Rosenberg PB. The fornix in mild cognitive impairment and Alzheimer’s disease. Front Aging Neurosci 2015;7 Preprint at 10.3389/fnagi.2015.00001. 10.3389/fnagi.2015.00001 PMC4301006 25653617

[38] Rajagopalan V et al. A basic introduction to Diffusion Tensor Imaging mathematics and Image Processing steps. Brain Disord Ther 06, (2017).

[39] Lissek S, Glaubitz B, Schmidt-Wilcke T, Tegenthoff M (2016). Hippocampal Context Processing during Acquisition of a predictive Learning Task is Associated with Renewal in extinction recall. J Cogn Neurosci.

[40] Lissek S, Golisch A, Glaubitz B, Tegenthoff M (2017). The GABAergic system in prefrontal cortex and hippocampus modulates context-related extinction learning and renewal in humans. Brain Imaging Behav.

[41] Lissek S, Klass A, Tegenthoff M (2022). NMDA receptor-mediated processing in inferior frontal gyrus facilitates acquisition and extinction learning and strengthens renewal. Neurobiol Learn Mem.

[42] Chattarji S, Tomar A, Suvrathan A, Ghosh S, Rahman MM. Neighborhood matters: divergent patterns of stress-induced plasticity across the brain. Nat Neurosci 2015 18:(10)1364–1375. 10.1038/nn.4115 26404711

[43] Cook SC, Wellman CL (2004). Chronic stress alters dendritic morphology in rat medial prefrontal cortex. J Neurobiol.

[44] McEwen BS (2015). Mechanisms of stress in the brain. Nat Neurosci.

[45] Lissek S, Klass A, Tegenthoff M. Effects of noradrenergic stimulation upon context-related extinction learning performance and BOLD activation in hippocampus and prefrontal cortex differ between participants showing and not showing renewal. Front Behav Neurosci. 2019;13. 10.3389/fnbeh.2019.00078 PMC6491890 31105536

[46] Kinner VL, Merz CJ, Lissek S, Wolf (2016). O. T. Cortisol disrupts the neural correlates of extinction recall. NeuroImage.

[47] Leemans A, Jeurissen B, Sijbers J, Jones DK. ExploreDTI: a graphical toolbox for processing, analyzing, and visualizing diffusion MR data. Proc Intl Soc Mag Reson Med. 2009:3537.

[48] Basser PJ, Pajevic S, Pierpaoli C, Duda J, Aldroubi A (2000). Vivo Fiber Tractography using DT-MRI data. Magn Reson Med.

[49] Catani M, de Thiebaut M (2008). A diffusion tensor imaging tractography atlas for virtual in vivo dissections. Cortex.

[50] Figley CR et al. Potential pitfalls of using Fractional Anisotropy, Axial Diffusivity, and Radial Diffusivity as biomarkers of cerebral White Matter Microstructure. Front Neurosci 2022;15:799576. 10.3389/fnins.2021.799576 PMC8795606 35095400

[51] Aung WY, Mar S, Benzinger TL (2013). Diffusion tensor MRI as a biomarker in axonal and myelin damage. Imaging Med.

[52] Song SK (2003). Diffusion tensor imaging detects and differentiates axon and myelin degeneration in mouse optic nerve after retinal ischemia. NeuroImage.

