# Supplementary material for: Microstructural differences in the cingulum and the inferior longitudinal fasciculus are associated with (extinction) learning
Source: BMC Psychol. 2024 Jun 3;12:324. doi: 10.1186/s40359-024-01800-y (PMC11149371; doi:10.1186/s40359-024-01800-y)
Supplement: Supplementary file 1 — Supplementary Material 1. [file 40359_2024_1800_MOESM1_ESM.docx]

| **Supplement 1.** Overview of microstructural properties of WMT of interest. We calculated mean and SD of FA, RD, MD, and λ_1-3_ of all participants. | | | | | | | | | | | | | | |
| --- | --- | --- | --- | --- | --- | --- | --- | --- | --- | --- | --- | --- | --- | --- |
|  |  | **ILF** | | **Temporal CNG** | | **Superior CNG** | | **IFOF** | | **UNC** | | **FX** | |  |
|  |  | *Left* | *Right* | *Left* | *Right* | *Left* | *Right* | *Left* | *Right* | *Left* | *Right* | *Left* | *Right* |  |
| **FA*** | | 0.49  ± 0.032 | 0.48  ± 0.029 | 0.43  ± 0.019 | 0.41  ± 0.021 | 0.48  ± 0.023 | 0.50  ± 0.023 | 0.51  ± 0.023 | 0.50  ± 0.022 | 0.45  ± 0.025 | 0.43  ± 0.02 | 0.40  ± 0.023 | 0.40  ± 0.024 |  |
| **RD *** | | 5.4x10^-4^  ±  3.61x10^-5^ | 5.6x10^-4^  ±  3.51x10^-5^ | 5.9x10^-4^  ±  2.33x10^-5^ | 6.2x10^-4^  ±  3.04x10^-5^ | 5.2x10^-4^  ±  2.47x10^-5^ | 5.1x10^-4^  ±  2.45x10^-5^ | 5.3x10^-4^  ±  2.77x10^-5^ | 5.4x10^-4^  ±  3.13x10^-5^ | 5.4x10^-4^  ±  3.17x10^-5^ | 5.9x10^-4^  ±  3.96x10^-5^ | 1.02x10^-3^  ±  1.11x10^-4^ | 1.01x10^-3^  ±  8.74x10^-5^ |  |
| **MD ^*^** | | 7.7x10^-4^  ±  2.89x10^-5^ | 7.9x10^-4^  ±  2.51x10^-5^ | 7.9x10^-4^  ±  2.21x10^-5^ | 8.1x10^-4^  ±  2.94x10^-5^ | 7.4x10^-4^  ±  2.15x10^-5^ | 7.4x10^-4^  ±  2.01x10^-5^ | 7.8x10^-4^  ±  2.44x10^-5^ | 7.9x10^-4^  ±  2.93x10^-5^ | 7.5x10^-4^  ±  2.83x10^-5^ | 8.0x10^-4^  ±  3.5x10^-5^ | 1.33x10^-3^  ±  1.24x10^-4^ | 1.32x10^-3^  ±  9.33x10^-5^ |  |
| **λ1 ^*^** | | 1.23x10^-3^  ±  4.04x10^-5^ | 1.26x10^-3^  ±  2.72x10^-5^ | 1.20x10^-3^  ±  3.19x10^-5^ | 1.21x10^-3^  ±  3.76x10^-5^ | 1.16x10^-3^  ±  3.01x10^-5^ | 1.20x10^-3^  ±  3.01x10^-5^ | 1.27x10^-3^  ±  3.25x10^-5^ | 1.28x10^-3^  ±  3.63x10^-5^ | 1.15x10^-3^  ±  3.32x10^-5^ | 1.20x10^-3^±  3.4x10^-5^ | 1.95x10^-3^  ±  1.61x10^-4^ | 1.93x10^-3^  ±  1.13x10^-4^ |  |
| **λ2 ^*^** | | 6.6x10^-4^  ±  3.48x10^-5^ | 6.7x10^-4^  ±  3.71x10^-5^ | 6.7x10^-4^  ±  2.37x10^-5^ | 7.0x10^-4^  ±  3.16x10^-5^ | 6.1x10^-4^  ±  2.26x10^-5^ | 6.0x10^-4^  ±  2.47x10^-5^ | 6.3x10^-4^  ±  2.78x10^-5^ | 6.5x10^-4^  ±  3.08x10^-5^ | 6.2x10^-4^  ±  3.11x10^-5^ | 6.8x10^-4^  ±  4.1x10^-5^ | 1.09x10^-4^  ±  1.04x10^-4^ | 1.08x10^-4^  ±  8.57x10^-5^ |  |
| **λ3 ^*^** | | 4.3x10^-4^  ±  3.98x10^-5^ | 4.5x10^-4^  ±  3.47x10^-5^ | 5.0x10^-4^  ±  2.59x10^-5^ | 5.4x10^-4^  ±  3.12x10^-5^ | 4.3x10^-4^  ±  2.87x10^-5^ | 4.3x10^-4^  ±  2.55x10^-5^ | 4.4x10^-4^  ±  2.98x10^-5^ | 4.3x10^-4^  ±  3.41x10^-5^ | 4.6x10^-4^  ±  3.34x10^-5^ | 5.0x10^-4^  ±  4.06x10^-5^ | 9.5x10^-4^  ±  1.16x10^-4^ | 9.4x10^-4^  ±  9.01x10^-5^ |  |

*mm^2^/s
